# Reliability and prognostic value of radiomic features are highly dependent on choice of feature extraction platform

**DOI:** 10.1007/s00330-020-06957-9

**Published:** 2020-06-01

**Authors:** Isabella Fornacon-Wood, Hitesh Mistry, Christoph J. Ackermann, Fiona Blackhall, Andrew McPartlin, Corinne Faivre-Finn, Gareth J. Price, James P. B. O’Connor

**Affiliations:** 1 Division of Cancer Sciences, University of Manchester, Manchester, UK; 2 Department of Medical Oncology, Spital STS AG, Thun, Switzerland; 3 Department of Medical Oncology, The Christie Hospital NHS Foundation Trust, Manchester, UK; 4 Department of Clinical Oncology, The Christie Hospital NHS Foundation Trust, Manchester, UK; 5 Department of Diagnostic Radiology, The Christie Hospital NHS Foundation Trust, Manchester, UK

**Keywords:** Reliability of results, Biomarkers, Prognosis, Tomography, x-ray computed, Translation

## Abstract

**Objective:**

To investigate the effects of Image Biomarker Standardisation Initiative (IBSI) compliance, harmonisation of calculation settings and platform version on the statistical reliability of radiomic features and their corresponding ability to predict clinical outcome.

**Methods:**

The statistical reliability of radiomic features was assessed retrospectively in three clinical datasets (patient numbers: 108 head and neck cancer, 37 small-cell lung cancer, 47 non-small-cell lung cancer). Features were calculated using four platforms (PyRadiomics, LIFEx, CERR and IBEX). PyRadiomics, LIFEx and CERR are IBSI-compliant, whereas IBEX is not. The effects of IBSI compliance, user-defined calculation settings and platform version were assessed by calculating intraclass correlation coefficients and confidence intervals. The influence of platform choice on the relationship between radiomic biomarkers and survival was evaluated using univariable cox regression in the largest dataset.

**Results:**

The reliability of radiomic features calculated by the different software platforms was only excellent (ICC > 0.9) for 4/17 radiomic features when comparing all four platforms. Reliability improved to ICC > 0.9 for 15/17 radiomic features when analysis was restricted to the three IBSI-compliant platforms. Failure to harmonise calculation settings resulted in poor reliability, even across the IBSI-compliant platforms. Software platform version also had a marked effect on feature reliability in CERR and LIFEx. Features identified as having significant relationship to survival varied between platforms, as did the direction of hazard ratios.

**Conclusion:**

IBSI compliance, user-defined calculation settings and choice of platform version all influence the statistical reliability and corresponding performance of prognostic models in radiomics.

**Key Points:**

*• Reliability of radiomic features varies between feature calculation platforms and with choice of software version.*

*• Image Biomarker Standardisation Initiative (IBSI) compliance improves reliability of radiomic features across platforms, but only when calculation settings are harmonised.*

*• IBSI compliance, user-defined calculation settings and choice of platform version collectively affect the prognostic value of features.*

**Electronic supplementary material:**

The online version of this article (10.1007/s00330-020-06957-9) contains supplementary material, which is available to authorized users.

## Introduction

There is considerable current interest in calculating features from medical images using high-throughput methods and then relating these features to clinical endpoints [1, 2]. This approach has been termed ‘radiomics’. The principal hypothesis is that medical images contain information beyond that identified readily by traditional radiological examination, and that this information can be extracted through advanced image analysis. Since imaging plays a key role in cancer diagnosis, treatment and follow-up, radiomics provides potential non-invasive and inexpensive methods for developing biomarkers for prognosis and/or prediction in oncology.

The potential value of radiomic biomarkers has been well documented [1, 3], but recent literature have highlighted potential barriers to the translation of radiomics into useful decision-making tools [4, 5]. For example, studies have demonstrated that radiomic features can be heavily influenced by scanner acquisition and reconstruction parameters [6, 7] or inter-observer variability in defining target lesions [8], both of which influence model performance [9, 10].

One critical aspect of the radiomics workflow that remains relatively unexamined is the implementation of the software platforms used to calculate radiomic features. Many radiomic software platforms are reported in the literature, ranging from in-house developments [11], to open-source [12–14], freeware [15] and commercial offerings [16]. With in-house and commercial products, the source code for calculating features is not always publically available. This can prevent comparison of results between studies in the literature. This is contrary to current moves towards an open-science approach in ‘big data’ analyses and in artificial intelligence, where open-source and freeware developers publish feature definitions alongside software code, including the values chosen for any calculation settings, and the user-defined free parameters that are required for the calculation of some features [17].

Several studies have previously demonstrated that features can vary when calculated in different software platforms [18–20]. The Image Biomarker Standardisation Initiative (IBSI) is an international collaboration developed to help standardise radiomic feature calculation and has provided a framework to deliver practical solutions to this problem [21]. The IBSI has made recommendations concerning feature calculation, standardised feature definition and nomenclature. It has also provided a digital phantom with benchmark values to validate feature calculation platforms (to become IBSI-compliant) [22]. However, IBSI does not address calculation settings or evaluate versions of software.

In this article, we expand on this work by looking in three clinical datasets. We aimed to investigate the effects of IBSI compliance, harmonisation of calculation settings and choice of platform version on the statistical reliability of radiomic features and their corresponding ability to predict clinical outcome.

## Methods and materials

In this study, we evaluated three different clinical datasets using four different radiomic feature calculation platforms.

### Patient data

Data analysis was performed following institutional board approval and was compliant with UK research governance (ref. 17/NW/0060). We examined three datasets:

1. One hundred eight radiotherapy planning contrast-enhanced CT scans from patients with oropharyngeal head and neck (H&N) cancer treated with either chemo-radiotherapy or radiotherapy alone at The Christie NHS Foundation Trust, Manchester, UK.
2. Thirty-seven radiotherapy planning contrast-enhanced CT scans from a cohort of patients with small-cell lung cancer (SCLC) who had been enrolled in the CONVERT trial [23], acquired in nine different institutions (Supplementary Material A).
3. Forty-seven diagnostic contrast-enhanced CT scans from a cohort of patients with stage 4 non-small-cell lung cancer (NSCLC) cancer treated with first-line immunotherapy at The Christie NHS Foundation Trust, Manchester, UK.

The gross tumour volume, the extent of the visible tumour on the CT scan, was extracted from the radiotherapy structure set for both the H&N and SCLC cohorts. Original contours were drawn by the treating physician using the Pinnacle3 Treatment Planning system (versions 8.0, 9.0, 9.8 or 16.0, Philips Healthcare) and used as the analysis region of interest (ROI). Twelve H&N and 10 SCLC patients did not have contrast due to poor renal function or IV access. For the NSCLC dataset, ROIs were drawn by a thoracic oncologist (C.A.; 5 years’ experience) using the same Pinnacle software (version 9.8). ROIs were checked by a board-certified radiologist J.O.C.: 14 years’ experience). Full details of patient cohorts, image acquisition and reconstruction are detailed in Supplementary Tables 1 and 2.

### Radiomic software platform selection

To our knowledge, 14 different radiomics software platforms are reported in the literature (Table 1) [12–15, 24–29]. Four of these software platforms are freely available, used widely in the literature and have mathematical equations documented to sufficient detail to understand the basis for their analysis.

**Table 1:** Details of various software packages available for radiomic feature calculation. The listed number of citations are those that cite the initial publication introducing the platform according to PubMed (search on 30/01/2020)

Software	Year of publication	Citations	IBSI-compliant?	Free?	Open source?	Feature sets calculated	Mathematical equations documented?
MaZda [24]	2009	366	×	✓	×	Shape, intensity and texture	×
Chang-Gung Image Texture Analysis (CGITA) [25]	2014	65	×	✓	✓	Intensity and texture	×
IBEX [13]	2015	134	×	✓	✓	Shape, intensity and texture	✓
Moddicom [26]	2015	13	×	✓	✓	Shape, intensity and texture	×
PyRadiomics [14]	2017	324	✓	✓	✓	Shape, intensity and texture	✓
LIFEx [15]	2018	84	✓	✓	×	Shape, intensity and texture	✓
Quantitative Image Feature Engine (QIFE) [27]	2018	13	×	✓	✓	Shape, intensity and texture	×
CERR [12]	2018	25	✓	✓	✓	Shape, intensity and texture	✓
MITK Phenotyping [28]	2019	6	✓	✓	✓	Shape, intensity and texture	✓
RaCat [29]	2019	4	✓	✓	✓	Shape, intensity and texture	×
PORTS v.1.1 matlab software (www.ncihub.org/resources/1663)	Not published	Not published	×	✓	✓	Intensity and texture	✓
MatLab package (www.github.com/mvallieres/radiomics)	Not published	Not published	✓	✓	✓	Shape, intensity and texture	✓
TexRad	Not published	Not published	Unknown	×	×	Unknown	Unknown
Oncoradiomics	Not published	Not published	Unknown	×	×	Unknown	Unknown

For all of the study, we used the latest version of the following platforms: LIFEx v5.47 [15], IBEX v1.0 beta [13], PyRadiomics v2.2.0 [14] and the Computational Environment for Radiological Research (CERR) commit a1c8181 (05/09/2019) available at https://github.com/cerr/CERR [12]. Notably, LIFEx, PyRadiomics and CERR claim compatibility with the IBSI standard, whereas IBEX does not (Table 1).

For the comparison between software versions, we used LIFEx v5.1, CERR commit 50530f7 (29/08/2019) and PyRadiomics v2.1.2. IBEX has only released one version.

### Feature calculation

**Fig. 1:**
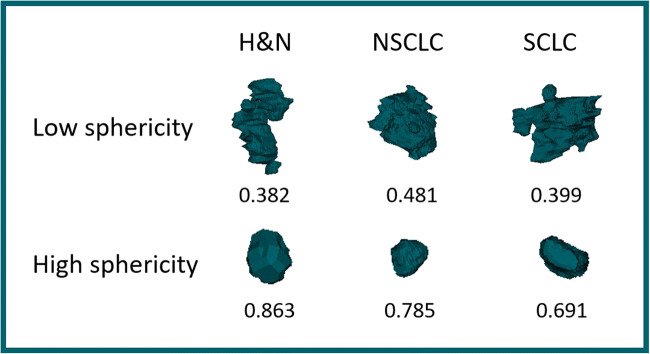
Example tumours and corresponding values for the feature ‘sphericity’ from each dataset

We analysed radiomic features common to the four software platforms. These 17 features included three shape parameters, four intensity feature, one histogram feature, six 3D grey level co-occurrence matrix (GLCM) features and three 3D neighbourhood grey tone difference matrix (NGTDM) features measuring ROI heterogeneity (Table 2; example of the shape feature ‘sphericity’ shown in Fig. 1). Since naming conventions for these features are not consistent across software (see Table 2), we used the feature names most closely in keeping with IBSI nomenclature, but simplified where appropriate. No image pre-processing was performed.

**Table 2:** Differences in naming conventions defined by the IBSI across the radiomic software. *ID*, inverse difference; *GLCM*, grey-level co-occurrence matrix; *HU*, Hounsfield Unit; *NGLDM*, neighborhood grey-level different matrix; *NGTDM*, neighboring grey tone difference matrix

Feature	IBSI terminology	LIFEx	IBEX	PyRadiomics	CERR
Volume	Volume (mesh) and volume (voxel counting)	Volume	Volume	Mesh volume and voxel volume	Volume
Sphericity	Sphericity	Sphericity	Sphericity	Sphericity	Sphericity
Area	Surface area (mesh)	Surface area	Surface area	Surface area	Surface area
Skewness	Discretised intensity skewness	Histogram skewness	Intensity histogram skewness	First-order skewness	Skewness
GLCM correlation	GLCM correlation	GLCM correlation	GLCM correlation	GLCM correlation	GLCM correlation
GLCM contrast	GLCM contrast	GLCM contrast = variance	GLCM contrast	GLCM contrast	GLCM contrast
GLCM angular Second moment	GLCM angular Second moment	GLCM energy = angular second moment	GLCM energy	GLCM joint energy	GLCM joint energy
GLCM joint entropy	GLCM joint entropy	GLCM entropy Log2 = joint entropy	GLCM entropy	GLCM joint entropy	GLCM joint entropy
GLCM difference average	GLCM difference average	GLCM dissimilarly	GLCM dissimilarly	GLCM difference average	Dissimilarity (difference average)
GLCM inverse difference	GLCM inverse difference	GLCM homogeneity = inverse difference	GLCM homogeneity	GLCM ID	GLCM inverse difference
NGTDM busyness	NGTDM busyness	NGLDM busyness	Neighbour intensity difference busyness	NGTDM busyness	NGTDM busyness
NGTDM coarseness	NGTDM coarseness	NGLDM coarseness	Neighbour intensity difference coarseness	NGTDM coarseness	NGTDM coarseness
NGTDM contrast	NGTDM contrast	NGLDM contrast	Neighbour intensity difference contrast	NGTDM contrast	NGTDM contrast
Minimum	Minimum intensity	Conventional HU minimum	Global Minimum	First-order minimum	Minimum
Maximum	Maximum intensity	Conventional HU maximum	Global maximum	First-order maximum	Maximum
Mean	Mean intensity	Conventional HU mean	Global mean	First-order mean	Mean
Standard deviation	Not defined (variance is defined)	Conventional HU standard deviation	Global standard deviation	First-order standard deviation	Standard deviation

The absolute numerical value of some radiomic features depend heavily on choice of default or user-defined settings. For example, the number of bins used to discretise image intensities do not have consistent default values across the platforms (see Table 3). Therefore, as well as performing inter-platform comparison of the results from different platforms, we also investigated the effect harmonising these parameters to common values. The harmonised calculation settings are presented in Table 3. Differences between platforms are detailed in Supplementary Material B.

**Table 3:** Default calculation settings for each software platform along with the harmonised settings used in this study

Calculation settings	LIFEx	IBEX	PyRadiomics	CERR	Harmonised settings (this study)
Histogram					
Number of grey levels	400	256	Bin width 25	Bin width 25	64
Lower bound	− 1000	0	Minimum	0	Minimum
Upper bound	3000	4096	Maximum	500	Maximum
GLCM					
Number of grey levels	400	100	Bin width 25	Bin width 25	64
Lower bound	− 1000	0	Minimum	0	Minimum
Upper bound	3000	2100	Maximum	500	Maximum
Directions	13	13	13	4	13
Offset	1	1, 4 and 7	1	1	1
Symmetric	Yes	Yes	Yes	Yes	Yes
NGTDM					
Number of grey levels	400	256	Bin width 25	Bin width 25	64
Lower bound	− 1000	0	Minimum	0	Minimum
Upper bound	3000	4096	Maximum	500	Maximum
Distance	1	2	1	1	1

### Statistical analysis

To assess the effect of software platform variation on the reliability of radiomic biomarkers, we calculated two-way mixed effect intraclass correlation coefficients (ICC) and their 95% confidence intervals (CIs) for each feature. The ICC quantifies the absolute agreement between features computed by each platform. The ICC estimates and CI were stratified to indicate poor (ICC CI < 0.5), moderate (0.5 < ICC CI < 0.75), good (0.75 < ICC CI < 0.9) and excellent (ICC CI > 0.9) reliability [30]. Negative ICC estimates and CI were truncated at zero.

To assess the effect of software platform variation on the relationship of radiomic biomarkers to clinical outcome, we applied univariable cox regression against overall survival in the H&N dataset for each feature in Table 2. We repeated this analysis for each software platform using both their default calculation settings and the harmonised settings. Feature values were normalised to uniform scale (mean 0, standard deviation 1) to permit relative comparison of effect sizes.

All statistical analyses were performed in R 3.5.2 [31] with packages *irr* v0.84 [32] and *survival* v2.44.1.1 [33].

## Results

### Poor radiomic biomarker reliability across software platforms is improved by IBSI standardisation

**Fig. 2:**
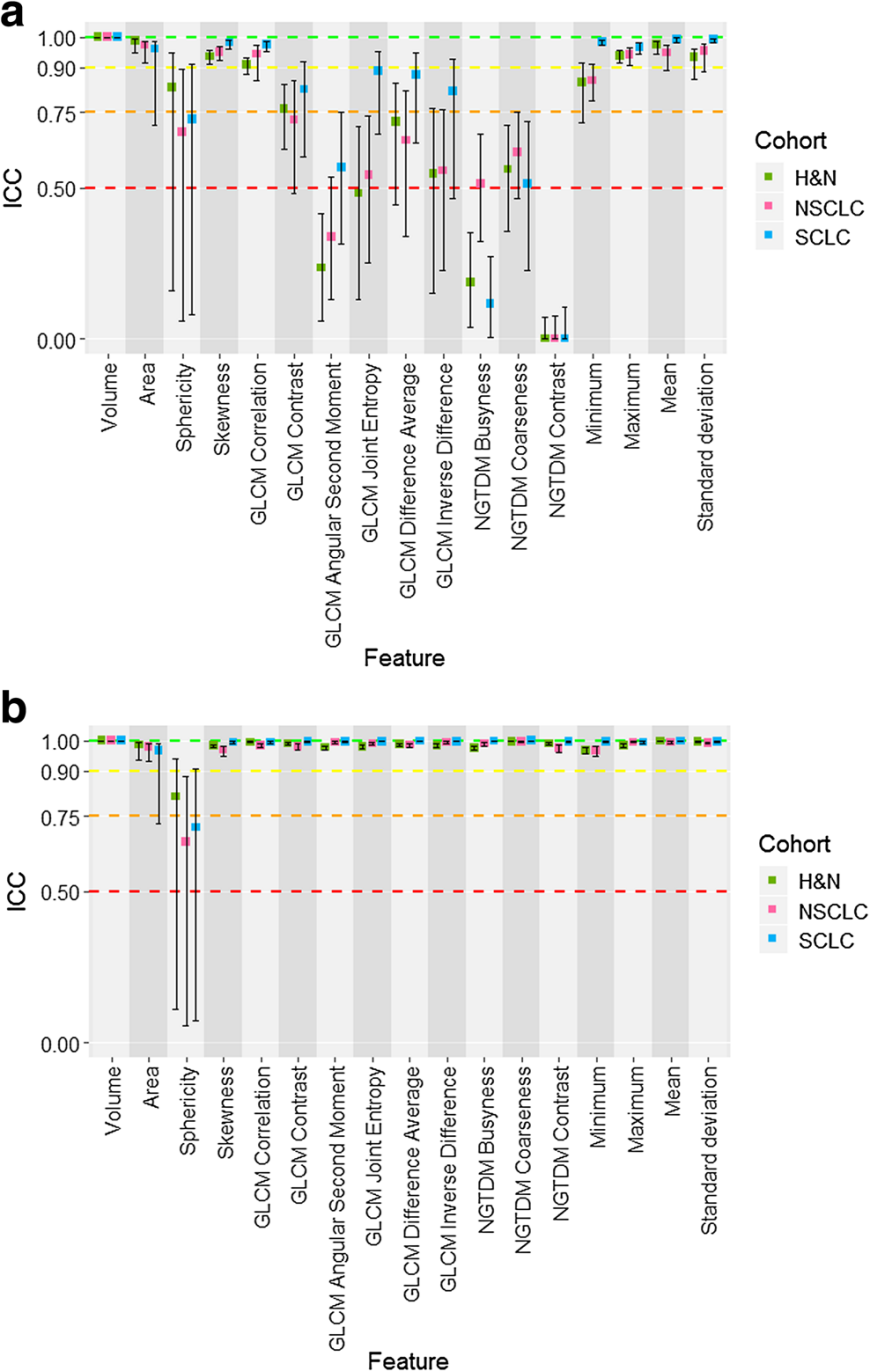
Boxplots of ICC estimates and CI for each cohort (H&N in green, NSCLC in pink, SCLC in blue) for all 17 features, showing the statistical reliability between the different software platforms. **a** ICC estimates and CI for all four software with harmonised calculation settings. **b** ICC estimates and CI for the three IBSI-compliant software with harmonised calculation settings (i.e. with IBEX excluded from analysis)

We assessed the statistical reliability between radiomic features calculated from four software platforms using harmonised calculation settings in three clinical datasets. The distribution of feature values across all platforms and cohorts is available in the Supplementary Data. In each case, ICC and confidence intervals were derived (Fig. 2a). Reliability between all four software was excellent (ICC CI > 0.9) in all datasets for only 4/17 features (volume, skewness, mean and maximum intensity). Reliability between software was poor (ICC CI < 0.5) in all datasets for 6/17 features (sphericity, some GLCM features and all NGTDM features). The other features had moderate or good reliability. Overall, the level of reliability for each individual feature was highly consistent across the three clinical datasets.

We repeated the analysis for only the IBSI-compliant software platforms, by removing IBEX data (Fig. 2b). This had a marked effect, with 15/17 features now showing excellent reliability across all datasets. Overall, these data show that the level of reliability across different radiomic biomarkers can vary substantially between different software platforms in the absence of IBSI-compliant standardisation. Once standardisation is adopted, this divergence is reduced substantially for most radiomic biomarkers.

### IBSI standardisation is only effective when calculation settings are harmonised

**Fig. 3:**
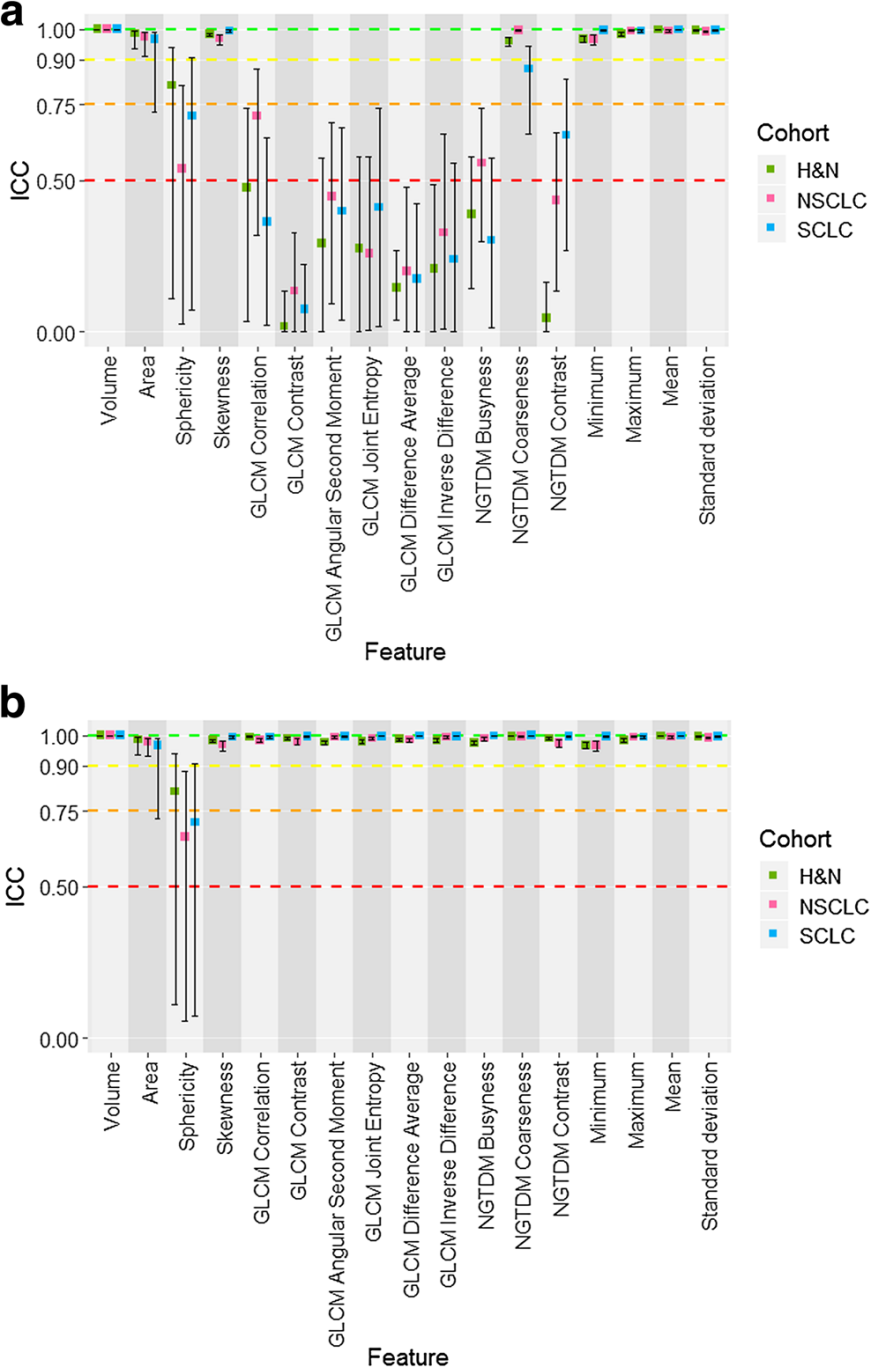
Boxplots of ICC estimates and CI for each cohort (H&N in green, NSCLC in pink, SCLC in blue) across all 17 features, showing the statistical reliability between the different software platforms. **a** ICC estimates and CI for the three IBSI-compliant software with default calculation settings (i.e. with IBEX excluded from analysis). **b** ICC estimates and CI for the three IBSI-compliant software with harmonised calculation settings (i.e. with IBEX excluded from analysis)

IBSI guidelines provide clear instructions and definitions for the process of image biomarker calculation. However, no recommendations are given for calculation settings. We evaluated the influence of using the default calculation settings versus harmonising them across software platforms using the three IBSI-compliant software platforms (Fig. 3a). Reliability was excellent for only 6/17 features (volume, skewness, standard deviation and mean, minimum, maximum intensity) when default calculation settings were used, despite all software being IBSI-compliant. In distinction, 10/17 features (sphericity, all six GLCM-based features and all three NGTDM-based features) had poor reliability across all three datasets.

Once calculation settings were harmonised, the reliability reverted to that seen for IBSI-compliant software (Fig. 3b). These data reveal the importance of these user-defined free parameters to the calculation of radiomic features. Without harmonisation of calculation settings, even IBSI-compliant platforms generate unreliable features, with the effect remarkably consistent across the three different tumour types and two different types of CT data (diagnostic and radiotherapy planning scans).

### Different versions of each software platform influence the statistical reliability of radiomic biomarkers

**Fig. 4:**
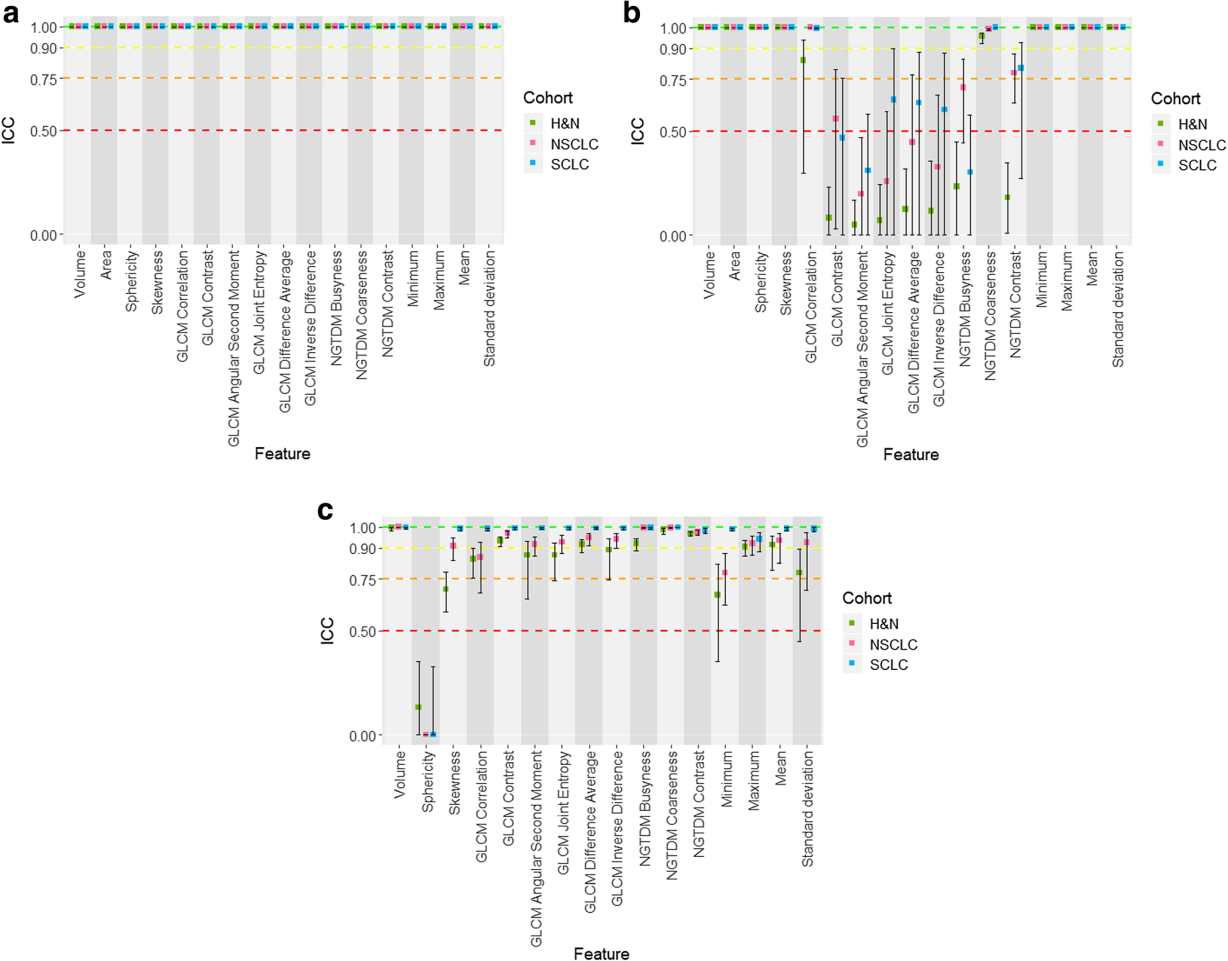
Boxplots of ICC estimates and CI for each cohort (H&N in green, NSCLC in pink, SCLC in blue) across all 17 features, showing the reliability between different versions of the same software platform. ICC estimates and CI are presented for (**a**) PyRadiomics version 2.2.0 versus 2.1.2 with harmonised calculation settings, (**b**) CERR commit a1c8181 versus 50530f7 with harmonised calculation settings and (**c**) LIFEx version 5.47 versus 5.1 with harmonised calculation settings (NB: area is not calculated in LIFEx version 5.1 and so does not appear in **c**)

Software platforms undergo frequent updates. We evaluated the effect of changing between software versions for all three IBSI-compliant platforms by calculating the ICC between the newer and older versions. PyRadiomics had excellent reliability for all features (Fig. 4a). CERR had a discretisation error in an older version (commit 50530f7 (29/08/2019) available at https://github.com/cerr/CERR) which affected texture features calculation (GLCM and NGTDM) (Fig. 4b). We identified this difference and, after making the developers aware, the source of error issue was discovered and corrected for the newest version, which is used in our full analysis.

Initial experiments showed that sphericity had poor reliability in all datasets, even when comparison was restricted to IBSI-compliant software platforms (Fig. 2b). Investigation traced this uncertainty to LIFEx (the sphericity values for CERR and PyRadiomics had ICC estimates with 95% CI of 0.996 to 0.999 (CI 0.992-1) for the three clinical datasets). Comparing the latest LIFEx release (5.1) with the development version used in this study (5.47) shows significant changes in sphericity (Fig. 4c). The minimum value calculation also changed between these versions with knock-on effect on dependent features, such as skewness, some GLCM features and standard deviation.

Taken together, these data reveal the importance of study authors reporting which software version was used for data analysis. The data also highlight the difficulty in comparing studies that initially appear to be similar to one another.

### Software platform and calculation settings affect the significance and direction of correlation of radiomic features to overall survival

**Fig. 5:**
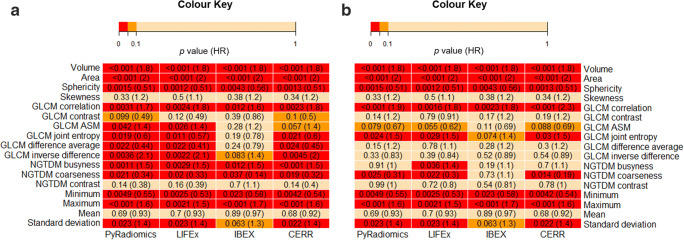
Heat-map of the *p* values (and associated hazard ratios) from univariable Cox regression for each radiomic feature, with harmonised calculation settings on the left (**a**) and default calculation settings on the right (**b**). Cells are colour-coded according to the following *p* value thresholds: *p* value < 0.05 (red), 0.05 < *p* value < 0.1 (orange) and *p* value > 0.1 light orange. *ASM*, angular second moment; *HR*, hazard ratio

We assessed how the choice of software platform and calculation settings influences the relationship of radiomic features to patient outcome. These analyses were performed in the largest of our clinical datasets (H&N cancer; *N* = 108). Overall survival was determined, with 28 patients dying within the follow-up period of 2.2 years. Univariable Cox regression results are presented for all 17 features with harmonised calculation settings and default calculation settings (Fig. 5).

The *p* values and associated hazard ratios for each feature when using harmonised calculation settings are presented in Fig. 5a. Eight features (volume, area, sphericity, GLCM correlation, NGTDM busyness, NGTDM coarseness, minimum and maximum) were significant at *p* < 0.05 in all four platforms. A further five features (GLCM angular second moment, GLCM joint entropy, GLCM difference average, GLCM inverse difference and standard deviation) were significant at *p* < 0.05 for the three IBSI-compliant software platforms but not in IBEX. When a given radiomic feature was deemed significant at the *p* < 0.05 threshold for multiple software platforms, the hazard ratios were generally in close agreement across the software platforms.

The *p* values and associated hazard ratios for each feature when using default calculation settings are presented in Fig. 5b. Since shape and most first-order features are not dependent on these parameters, they were unaffected by the changed calculation settings. Texture features, however, are dependent on the user-defined calculation settings and all became no longer significant at the *p* < 0.05 threshold, with the exception of GLCM correlation. Notably, IBEX diverged further from agreement with the three IBSI-compliant software platforms.

**Fig. 6:**
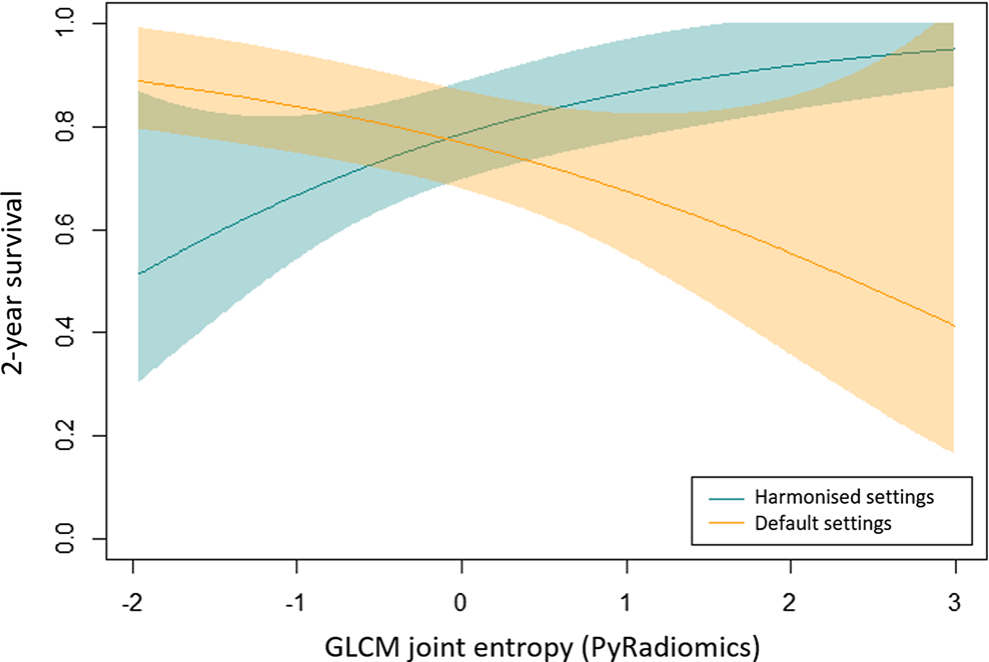
GLCM joint entropy (here calculated in PyRadiomics) against 2-year survival for patients with H&N cancer when calculated with harmonised settings (blue) and default settings (orange)

Of particular note, the hazard ratio for GLCM joint entropy changed from 0.56–0.59 (i.e. less than 1.0 and significant *p* value) when harmonised calculation settings were used to 1.5 (i.e. more than 1.0 and significant *p* value) when default calculation settings were used. Thus, significant correlations were detected that had opposing hazard ratio directions depending on choice of parameter input. This effect is shown clearly in Fig. 6, where the direction of the hazard ratio changed from protective to harmful. These data reveal that both IBSI compliance and calculation settings can affect the significance and direction of relationships between radiomic features and clinical outcome.

## Discussion

Radiomics has great potential to produce independent predictive biomarkers for personalised healthcare, particularly in the management of patients with cancer [2]. Many studies have been published describing prognostic and predictive radiomic signatures, but significant methodological limitations have hindered clinical translation of these techniques [34].

In this study, we investigated the importance of IBSI compliance, harmonising calculation settings and choice of platform version when using different radiomics calculation platforms. We tested how these factors affect the statistical reliability of features and showed how these factors also influence the relationship between radiomic biomarkers and clinical outcome (in this case, the overall survival).

Radiomic feature calculation is an important part of the radiomics workflow. Studies can use a variety of commercial or freely available software platforms to achieve this [16] or use in-house developed software. A study by Foy et al compared two in-house developed software to IBEX and found that for head and neck CT scans, histogram features had excellent reliability but GLCM features varied between poor and excellent reliability [18]. The software packages in that study were not IBSI-compliant.

Our study demonstrates the benefits of standardising feature calculation platforms according to the IBSI. Features calculated in IBSI-compliant software had greater statistical reliability than features calculated in non-compliant platforms, but only when calculation settings were also harmonised. The method of grey level discretisation has been shown to affect feature reproducibility within the same software platform [35, 36]. Our results both confirm these findings and extend the principle to all those user-defined parameters listed in Table 3, emphasising the need to harmonise calculation settings even when an IBSI-compliant platform is used. Results were highly consistent across three clinical datasets.

Our data has also highlighted the importance of inter-software comparison. By doing so, we identified potential errors in both the CERR and LIFEx code bases, leading to subsequent corrections and improved reliability. It is vital that investigators document the version and date of the software platform used in their study to ensure results are reproducible between institutions. Our data also highlight the benefits of open-source tools and the importance of the relevant scientific communities actively working with their developers to improve them.

Univariable survival analysis revealed substantial differences in prognostic power between supposedly similar features derived from different software platforms. We make three observations. Firstly, some features had significant association with H&N cancer overall survival in the IBSI-compliant software but not in IBEX. These findings concur with Liang et al who investigated two platforms and found differences in downstream clustering of known prognostic factors in patients with nasopharyngeal carcinoma [20]. Similar conclusions were drawn by Bogowicz et al who investigated this in PET scans of patients with H&N cancer [19]. Secondly, when only evaluating IBSI-compliant software, there was a divergence of feature to survival correlation between software platforms when calculation settings varied.

Thirdly, our study demonstrates that when different calculation settings are used, the relationship of significant features to survival can remain significant but the direction of that relationship (hazard ratio) can invert from protective to harmful. This effect may reflect that for some features, altering calculation settings radically alters the biophysical property being measured. In this study, there is no ground truth against which the ‘true’ direction of a feature can be established, but the data demonstrates the important role calculation settings play in selecting features for radiomic signatures.

There are several limitations to this study. Our inclusion criteria for feature calculation platforms that they are freely available, widely cited, and sufficiently well documented for analysis limited the number of assessed platforms to four, only one of which was not IBSI-compliant. There are also more features available in each of the software platforms that were not included in this study, as only features that were available across all four software platforms were analysed. The clinical datasets used were sufficiently large to evaluate ICC with CIs but the number of events only permitted univariable survival analysis of outcome. Lastly, LIFEx is a closed-source project, which precluded thorough investigation of the observed difference in sphericity calculation compared to other IBSI-compliant software.

In conclusion, this study has shown that use of IBSI-compliant radiomic feature calculation platforms appears to increase the statistical reliability of features. However, even IBSI-compliant platforms are affected strongly by user-defined calculation settings and changes between software versions. Future radiomics studies should be aware of potential differences between software platforms and ensure platforms used for radiomics studies are IBSI-compliant. Studies should ensure software version and user-defined parameters are clearly reported. Furthermore, the radiomics community should consider working towards a recommended set of harmonised calculation settings. Locking imaging biomarkers down in this way will improve the technical quality of data from subsequent studies, a vital step towards their translation into clinical decision-making tools [37].

## Electronic supplementary material

ESM 1 (DOCX 33 kb)

ESM 2 (CSV 11.2 kb)

ESM 3 (CSV 11.2 kb)

## Abbreviations

IBSI: Image biomarker standardisation initiative
ICC: Intraclass correlation coefficient
