# Supplementary material for: Reliability and prognostic value of radiomic features are highly dependent on choice of feature extraction platform
Source: Eur Radiol. 2020 Jun 1;30(11):6241–50. doi: 10.1007/s00330-020-06957-9 (PMC7553896; doi:10.1007/s00330-020-06957-9)
Supplement: Supplementary file 1 — (DOCX 33 kb) [file 330_2020_6957_MOESM1_ESM.docx]

**Supplementary**

Supplementary Table 1 Patient characteristics for the H&N, NSCLC and SCLC cohorts.

|  | H&N (n=108) | NSCLC (n=47) | SCLC (n=37) |
| --- | --- | --- | --- |
| Sex (n (%)) |  |  |  |
| Female | 26 (24.1) | 20 (42.6) | 15 (40.5) |
| Male | 82 (75.9) | 27 (57.4) | 22 (59.5) |
| Age at start of treatment (median [IQR]) | 61.50 [56.00, 67.00] | 68.50 [63.00, 73.00] | 60.00 [54.00, 66.00] |
| T stage (n (%)) |  |  |  |
| X | 0 (0) | 3 (6.4) | 0 (0) |
| 1 | 18 (16.7) | 5 (10.6) | 0 (0) |
| 2 | 41 (38.0) | 12 (25.5) | 9 (24.3) |
| 3 | 20 (18.5) | 7 (14.9) | 14 (37.8) |
| 4 | 26 (24.1) | 20 (42.6) | 12 (32.4) |
| Unknown | 3 ( 2.8) | 0 (0) | 2 (5.4) |
| N stage (n (%)) |  |  |  |
| X | 0 (0) | 2 (4.2) | 0 (0) |
| 0 | 40 (37.0) | 6 (12.8) | 9 (24.3) |
| 1 | 14 (13.0) | 2 (4.3) | 4 (10.8) |
| 2 | 46 (42.6) | 22 (46.8) | 18 (48.6) |
| 3 | 6 (5.6) | 15 (31.9) | 2 (5.4) |
| Unknown | 2 (1.9) | 0 (0) | 4 (10.8) |
| Performance status (n (%)) |  |  |  |
| 0 | 56 (51.9) | 5 (10.6) | 13 (35.1) |
| 1 | 40 (37.0) | 38 (80.9) | 24 (64.9) |
| 2 | 9 (8.3) | 4 (8.5) | 0 (0) |
| 3 | 3 (2.8) | 0 (0) | 0 (0) |
| HPV status (n (%)) |  |  |  |
| Negative | 14 (13.0) | NA | NA |
| Positive | 51 (47.2) | NA | NA |
| Unknown | 43 (39.8) | NA | NA |
| Chemotherapy (n (%)) |  |  |  |
| No | 50 (46.3) | 47 (100) | 0 (0) |
| Yes | 58 (53.7) | 0 (0) | 37 (100) |
| Radiotherapy prescribed dose (median [IQR]) | 65.40 [59.03, 66.00] | NA | 55.00 [45.00, 66.00] |

Supplementary Table 2 Image acquisition and reconstruction parameters for the SCLC, NSCLC and H&N CT datasets.

|  | H&N (n=108) | NSCLC (n=47) | SCLC (n=37) |
| --- | --- | --- | --- |
| Manufacturer, model | Philips, Brilliance Big Bore (n=94, 87.0%) | GE MEDICAL SYSTEMS, LightSpeed VCT (n=4, 8.5%) | GE MEDICAL SYSTEMS, HiSpeed CT/I (n=9, 24.3%) |
|  | SIEMENS, SOMATOM Definition AS (n=14, 13.0%) | GE MEDICAL SYSTEMS, Optima CT660 (n=3, 6.4%) | GE MEDICAL SYSTEMS, LightSpeed RT16 (n=4, 10.8%) |
|  |  | Philips, Ingenuity CT (n=1, 2.1%) | Philips, Brilliance Big Bore (n=3, 8.1%) |
|  |  | SIEMENS, SOMATOM Definition AS (n=38, 80.9%) | Philips, Gemini (n=1, 2.7%) |
|  |  | SIEMENS, SOMATOM Definition AS+ (n=1, 2.1%) | SIEMENS, Definition AS (n=16, 43.2%) |
|  |  |  | SIEMENS, Sensation Open (n=2, 5.4%) |
|  |  |  | SIEMENS, Spirit (n=2, 5.4%) |
| Slice thickness (mm) (median [IQR]) | 3 [3.00, 3.00] | 3 [3.00, 3.00] | 3.00 [3.00, 5.00] |
| Pixel spacing (mm) (median [IQR]) | 1.17 x 1.17 [1.17, 1.18] | 0.68 x 0.68 [0.63, 0.79] | 0.98 x 0.98 [0.94, 0.98] |
| Tube voltage (kVp) | 120 | 120 | 120 |
| Tube current (mAs) (median [IQR]) | 177.00 [99.00, 296.75] | 313.00 [267.00, 413.00] | 167.00 [140.00, 285.00] |
| Convolution kernel | B (n=94, 87.0%) | ['I70f', '2'] (n=20, 42.6%) | B (n=4, 10.8%) |
|  | B31f (n=14, 13.0%) | ['I70f', '3'] (n=2, 4.3%) | B31f (n=16, 43.2%) |
|  |  | B70f (n=1, 2.1%) | B31s (n=2, 5.4%) |
|  |  | B80f (n=16, 34.0%) | B41s (n=2, 5.4%) |
|  |  | C (n=1, 2.1%) | SOFT (n=9, 24.3%) |
|  |  | LUNG (n=7, 14.9%) | STANDARD (n=4, 10.8%) |

**Supplementary Material A**

The 37 radiotherapy planning contrast-enhanced CT scans from a cohort of patients with small cell lung cancer (SCLC) were acquired in nine different institutions, namely the Christie Hospital (Manchester, UK, N=28), Beatson Cancer Centre (Glasgow, UK, N=1), Bristol Haematology & Oncology Centre (Bristol, UK, N=1), Freeman Hospital (Newcastle-upon-Tyne, UK, N=1), Royal Marsden Hospital (London, UK, N=1), Institut Ste Catherine, Avignon, France (N=1), Centre Hospitalier Universitaire de Clermont-Ferrand (Clermont-Ferrand, France, N=2), Universiteit Gent (Gent, Belgium, N=1), Medical University of Gdansk (Gdansk, Poland, N=1).

**Supplementary Material B**

In LIFEx, features are calculated on the largest cluster of continuous voxels within the ROI only. To be able to compare results from LIFEx to results from PyRadiomics, IBEX and CERR, which use the whole ROI regardless of whether the voxels are continuous or not, only ROI’s with one cluster of voxels according to LIFEx were analyzed. This left 37 ROIs for comparison in the SCLC dataset, 108 ROIs in the H&N dataset and 47 ROIs in the NSCLC dataset.

In IBEX, the Hounsfield Units (HU) of the CT scan have 1000 added to them to ensure non-negative values, despite the fact that the lowest HU for a CT scan is -1014. Negative HU after this transformation are truncated at 0. To adjust for this in the minimum, maximum and mean comparison in Table 3, 1000 HU were taken from the IBEX values.

The IBSI define two methods for calculating the volume of a region of interest (ROI). The first is a mesh-based approach, where the surface of the ROI is represented as a mesh of triangles. The second method simply multiplies the volume of one voxel by the total number of voxels in the ROI. The voxel counting method does not handle partial volume effects at the ROI edge, which is particularly important for smaller volumes, and therefore the mesh-based approach is preferred [1]. PyRadiomics provide both options for volume calculation. In LIFEx, IBEX and CERR, volume is calculated using a voxel-counting approach.

The neighborhood grey tone difference matrix (NGTDM) as defined by the IBSI, PyRadiomics and CERR varies in its nomenclature. In IBEX it is known as the neighbor intensity difference matrix and in LIFEx as the neighborhood grey-level different matrix (NGLDM). The original definition of the NGTDM was developed by Amadasun and King [2]. The IBSI define the NGLDM as a different matrix entirely, originally developed by Sun and Wee [3], however the NGLDM in LIFEx is the same as the NGTDM as defined by the IBSI. Other than this, LIFEx correct their feature names to comply with the IBSI, for example “GLCM Homogeneity = Inverse Difference” since inverse difference is the IBSI-compliant feature definition.

**References**

1. Zwanenburg A, Leger S, Vallières M, Löck S Image biomarker standardisation initiative. arXiv Prepr arXiv161207003

2. Amadasun M, King R (1989) Texural Features Corresponding to Texural Properties. IEEE Trans Syst Man Cybern 19:1264–1274

3. Sun C, Wee WG (1983) Neighboring gray level dependence matrix for texture classification. Comput Vision, Graph Image Process 23:341–352
